# Association between thiol-disulfide hemostasis and transient tachypnea of the newborn in late-preterm and term infants

**DOI:** 10.1186/s12887-023-03936-z

**Published:** 2023-03-25

**Authors:** Mehmet Semih Demirtas, Huseyin Erdal, Fatih Kilicbay, Gaffari Tunc

**Affiliations:** 1 Department of Pediatrics, Faculty of Medicine, Aksaray University, Aksaray, Turkey; 2 Department of Medical Genetics, Faculty of Medicine, Aksaray University, Aksaray, Turkey; 3 Department of Neonatology, Faculty of Medicine, Pediatrics, Sivas Cumhuriyet University, Sivas, Turkey; 4 Department of Neonatology, Bursa Yüksek İhtisas Training and Research Hospital, Bursa, Turkey

**Keywords:** Transient Tachypnea of the newborn (TTN), Late preterm (34-36w), Thiol, Oxidative stress,, Newborn

## Abstract

**Background:**

Transient tachypnea of the newborn (TTN), which is the most common respiratory disease in the neonatal period, increases respiratory workload in newborns. We purposed to evaluate the oxidative stress (OS) status and thiol disulfide hemostasis in late preterm and term newborns with TTN in this study.

**Methods:**

The study was carried out in a single-centre neonatal intensive care unit to investigate the effect of continuous airway positive pressure (CPAP) on the oxidative system in newborns with TTN. Thiol (native and total) and disulfide levels, total antioxidant and oxidant status (TAS/TOS) and Oxidative stress index (OSI) levels were measured.

**Results:**

Total thiol levels measured before treatment was 429.5 (369.5–487) µmol/L in the late preterm group and 425 (370–475) µmol/L in the term group (*p* = 0.741). We found significant changes in TOS, OSI and TAS levels after CPAP treatment in the late preterm group (*p* < 0.001, *p* < 0.001, *p* = 0.012 respectively). It was also found that the disulfide level, which was 26.2 (19.2–31.7) before the treatment, decreased to 19.5 (15.5–28.75) after the treatment (*p* = 0.001) in late preterms.

**Conclusion:**

CPAP treatment reduced the OS status burden associated with TTN in neonates. The late preterm newborns with TTN are more affected by OS and increased OS levels decrease with CPAP treatment.

## Background

Transient tachypnea of the newborn (TTN), which is the most common respiratory disease in term and late preterm newborns, is a physiological lung parenchymal disorder caused by inadequate absorption or delayed clearance of fetal alveolar fluid [1]. It is known that fetal alveolar fluid, which fills the airways and alveoli in the intrauterine period, affects the lung mechanics positively by stretching the lungs. This fluid needs to be cleaned in order for effective gas exchange to take place after birth [2]. The main mechanism in TTN is thought to be delayed absorption of fetal alveolar fluid in the perinatal period. To compensate for this, tachypnea develops. If ventilation of the alveoli is further impaired, it leads to hypoxia [3]. Another responsible mechanism is that Na + transport, which is responsible for amiloride-sensitive Na^+^ channels (ENaC) in alveolarepithelial cells, is thought to play a role in reabsorption of fetal lung fluid. Since these channels are not activated or being immature at birth, the fluid in the lungs cannot be absorbed and leads to a decrease in respiratory functions of infants after birth. It has been shown that the expression of ENaC subunits is low in late preterm and term infants with transient neonatal tachypnea [1, 2, 4]. Decreased surfactant function has been also shown to contribute to the pathophysiology of TTN. In addition, the inability of the lung fluid to come out of the trachea due to the absence of high transpulmonary pressure caused by uterine contractions in normal vaginal deliveries in elective cesarean section is another mechanism shown in pathophysiology. [5]. Although TTN is considered to be a better clinical condition in terms of prognosis than other neonatal respiratory problems, it increases respiratory workload and stress in newborns [3, 5].

Thiol, which is one of the new parts of the oxidative system in metabolism and contains a sulfhydryl group (-SH), plays an important role in oxidative balance [6, 7]. Thiols in the structure of important amino acids containing sulphur, such as methionine, which is involved in the structure of many enzymatic reactions and hormones in the body, are the primary competition point for oxygen radicals. Oxidation of thiol groups with oxygen radicals also forms reversible disulfide bonds [6, 8]. The result of oxidation at the cellular level is the earliest findings of early protein oxidation. Increasing oxidative stress (OS) in the body causes disulfide formation by activating the oxidation of cysteine residues, and with the decrease of the OS load, the disulfide bonds are reduced to thiol again, therefore providing a dynamic thiol-disulfide balance [6]. Considering that growth and development in children continues until adulthood, the presence of thiol-disulfide homeostasis in mechanisms such as apoptosis detoxification and enzymatic reactions shows the importance of this system [6, 9].

Transient tachypnea of the newborn is an important neonatal respiratory problem due to its frequent occurrence and complications, especially in developing countries. We aimed to evaluate the relationship between thiol-disulfide hemostasis and also OS in late preterm and term newborns with TTN. The stabilization of this process can be evaluated biochemically, and the treatment can be arranged in the early period according to the patient's results.

## Methods

### Study design

This study was carried out an intervention study in a single 3rd level neonatal intensive care unit (NICU) at Sivas Cumhuriyet University, in order to examine the association between thiol/oxidative balance in late preterm and term patients with TTN. We conducted the study with a single-blind system: The samples of the patients were numbered by the researcher who collected the data, so that the other researcher who would work the samples was prevented from knowing the patient data.

### Study population

**Fig. 1:**
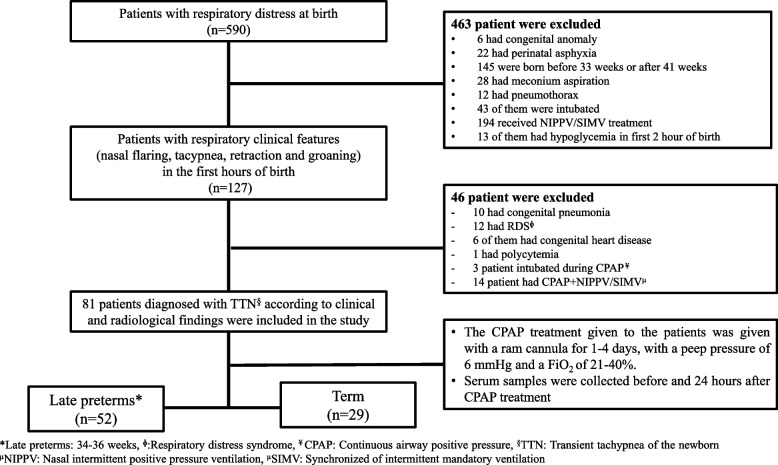
Flow diagram of the study

Patients who were born in our hospital unit and had symptoms of nasal flaring, tachypnea, retraction and groaning within the first few hours after birth were followed up with respiratory distress. Prenatal and perinatal detailed anamnesis was obtained from the parents of the patients. Chest x-rays, complete blood count (CBC), blood gas and CRP were organised from all patients. Neonates with delayed transition which resolves within six hours of birth, pneumothorax, respiratory distress syndrome (RDS), congenital diaphragmatic hernia, aspiration and pneumonia which are the other causes of respiratory distress was excluded with radiological imaging (chest x-ray). TTN was diagnosed based on clinical features (nasal flaring, tachypnea, retraction and groaning) and radiological features (diffuse streaks of perihilar interstitial opacities, fluid in the interlobar fissures, increased aeration, flattening in the diaphragm) in patients with respiratory distress who excluded other respiratory problems (Fig. 1). Hospitalized in the NICU with the diagnosis of TTN between March 2021 and January 2022; A total of 81 patients, 29 term (37–40 weeks) and 52 late preterm (34-36w) patients, were selected among 127 patients and included in the study. A total of 46 patients who were followed up with respiratory distress and had conditions such as RDS, congenital pneumonia, intubated, and congenital heart defects were excluded from the study. The inclusion and exclusion criteria of the study are given in Fig. 1 in detail. The severity of respiratory distress in patients after birth was evaluated with the Downes score [10].

### Data collection

Newborns born in our hospital, hospitalized in the NICU with TTN on the specified dates and treated with CPAP within the first 2 h after birth were included in the study. Patients who were intubated other than CPAP therapy or followed in another mode of NIPPV/SIMV were excluded from the study. The CPAP treatment given to the patients was given with a ram cannula for 1–4 days, with a peep pressure of 6 mmHg and a FiO2 of 21–40%. Hemogram (CBC), blood gas, C-reactive protein (CRP), aspartate amino transferase (AST), alanine amino transferase, creatinine, sodium (Na), potassium (K^+^), calcium (Ca), total bilirubin, direct bilirubin, direct coombs were taken from each patient before CPAP treatment. Serum samples (3 cc of blood) was collected in an EDTA blood tube before and 24 h after the end of CPAP treatment, centrifuged at 1500xrpm for 10 min. Serum samples obtained after this process were maintained at -80 °C until the study day for biochemical analysis. Serum samples were transported in accordance with the cold chain transport rules of biological material.

### Measurements of thiol–disulfide hemostasis

Before and after the treatment, OS parameters as disulfide and thiol (native/total) values were studied and their ratios were calculated by spectroscopic measurement of disulfide, native and total thiol levels [6].

### Measurement of plasma TOS, TAS and OSI

Plasma total oxidant status (TOS) levels (µmol H_2_O_2_ eq. /L) are expressed by spectrophotometric measurement of the sample formed as a result of the oxidants in the blood [8], oxidizing the ferrous ion-o-dianisidine complex to an iron ion and strengthening this reaction with glycerol molecules. Plasma Total Antioxidant Status (TAS) levels (µmol Trolox eqv. /L) are also a method based on the principle of measuring ABTS^+^ (2,2′-Azino-bis (3-ethylbenzthiazoline-6-sulfonic acid) cation after the reaction of H_2_O_2_ and ABTS described by Erel [8, 11].

In the calculation of oxidative stress index, firstly the TAS unit was converted to mol/L and the OSI unit was obtained with TOS/TAS [8].

### Statistical analysis

The data obtained was considered with descriptive statistics (arithmetic mean, median, interquartile ranges, percentage distributions). When comparing the mean between groups, first of all, the suitability for normal distribution was evaluated with the Shapiro Wilk and Kolmogorov Smirnov test. When comparing the mean of two independent quantitative groups, the Mann–Whitney U Test was used when non-parametric conditions are met. The Wilcoxon signed rank test was used in the use of two dependent quantitative data. Chi-square or Fisher’s exact test was used when comparing the percentage distributions of qualitative data. Spearman Correlation analysis was used for the data conforming to the normal distribution for the relationship between quantitative data. SPSS 22 program will be used in the analysis of the data, and a *p* < 0.05 level will be considered significant.

## Ethics

In the present study, informed consent was obtained from the parents of the patients after the procedure was explained within the ethical framework in accordance with the Declaration of Helsinki. Ethical approval was obtained from ethical committee of Sivas Cumhuriyet University with the number of 2022/01–07 in order to carry out the study.

## Results

The study conducted with 81 newborns who receiving CPAP treatment. The newborns in the study were examined in 2 groups as 29 term (≥ 37–41 weeks) and 52 late terms (≥ 34–36 weeks). There was a significant difference in birth weights between the two groups (p < 0.001). We didn’t find any difference between the two groups in terms of gender, Downes’ score, 1st and 5th. min. APGAR scores, ventilation times, mode of delivery and hemogram (CBC) parameters (*p* > 0.05) (Table 1).

**Table 1:** Mean blood gas, CBC parameters and patients’ demographic features

Parameters	Term (*n* = 29)	Late Preterm (*N* = 52)	Z	p
Birth Weight (gram)	3275 (2840–3645)	2160.5 (2015.5–2425)	**-**6.103	** < 0.001**
Gender n (%)				
Male	16 (55.2%)	23 (44.2%)		0.345^*^
Female	13 (44.8%)	29 (55.8%)		
Mode of Delivery n (%)				
NVD^¥^	4 (13.8%)	2 (3.8%)		0.180^*^
C/S^µ^	25 (86.2)	50 (96.2%)		
Downes’ score	5 (2–7)	6.5 (5–7)	-1.593	0.111
Ventilation Time (day)	2 (2–3)	2 (2–3)	-0.802	0.422
1st. min. APGAR score	7 (6–8)	7 (5.2–8)	-1.034	0.301
5th. min. APGAR score	9 (8–9)	8 (7.25–9)	-1.162	0.245
Hb (g/dl)	18 (16–19.68)	17 (16.05–19)	-0.554	0.580
Wbc (× 10^9^/l)	11.72 (9.1–14.5)	15.8 (11.8–19.3)	-2.66	**0.008**
Plt (× 10^9^/l)	278 (225.5–361)	276 (231–318.5)	-0.286	0.775
Crp (mg/L)	1.76 ± 3.06	1.25 ± 1.47	-0.094	0.925
pH	7.36 ± 0.5	7.34 ± 0.6	-0.387	0.699
pCO2 (mmHg)	43 (38- 50.5)	43 (39.75–48)	-0.040	0.968
pO_2_ (mmHg)	45 (34–49)	48 (35–51)	-0.535	0.592
Lactat	2.8 (1.75–3.8)	2.15 (1.8–2.56)	-1.310	0.191
HCO_3_ (mEq/L)	21.6 (19–23)	22 (20–23.9)	-0.722	0.470

Data are stated as median with interquartile range values

^*^Fisher’s exact test

^¥^Normal vaginal delivery, *C/S* Cesarean section. Mann–Whitney U test or Fisher’s exact test was used for data analyses

Total thiol levels measured before treatment was 429.5 (369.5–487) µmol/L in the late preterm group and 425 (370–475) µmol/L in the term group (*p* = 0.741). The disulfide level was found to be 26.25 (19.25–31.75) in the late preterm group and 27.5 (24.75–35) in the term group (*p* = 0.135). No significant relationship was found in other OS parameters examined (Table 2).

**Table 2:** Evaluation of oxidative stress parameters before and after CPAP treatment

Parameters	Before CPAP	Median Interquartile range	Z	p	After CPAP	Median Interquartile range	Z	p
Native thiol (µmol/L)	LPs*	386 (309–425.8) 375 (305.5–416)	-0.532	0.595	LPs^*^	367 (299–461.7) 361 (297–424.5)	-0.286	0.775
	Term				Term			
Total thiol (µmol/L)	LPs	429.5 (369.5–487) 425 (370–475)	-0.330	0.741	LPs	398 (346–503.5) 426 (346–480)	-0.163	0.871
	Term				Term			
Disulfide (µmol/L)	LPs	26.25 (19.25–31.75) 27.5 (24.75–35)	-1.49	0.135	LPs	19.5 (15.5–28.7) 18.5 (15–28.5)	-0.579	0.563
	Term				Term			
Disulfide/Native thiol (%)	LPs	7.44 (6.33–9.68) 6.93 (5.64–9)	-1.96	0.051	LPs	5 (3.7–7.6) 4.7 (3.1–8.35)	-0.435	0.664
	Term				Term			
Disulfide/Total thiol (%)	LPs	6.66 (5.20–8.57) 6.47 (5.62–8.11)	-1.95	0.052	LPs	4.5 (3.4–6.65) 4.3 (2.9–7.15)	-0.449	0.653
	Term				Term			
Native thiol/Total thiol (%)	LPs	88.25 (85.36–90.57) 87.05 (83.78–88.77)	-1.95	0.052	LPs	91 (86.7–93) 91.4 (85.6–94.2)	-0.425	0.671
	Term				Term			
TAS (mmol Trolox equiv. /lt)	LPs	1.52 (1.29–1.82) 1.49 (1.29–1.72)	-0.567	0.571	LPs	1.75 (1.53–2.12) 1.70 (1.56–2.05)	-1.28	0.201
	Term				Term			
TOS (µmol H_2_O_2_ equiv. /lt)	LPs	8.5 (6.42–9.48) 7.1 (6.7–8.9)	-0.917	0.359	LPs	5.23 (3.61–7.1) 5.1 (3.60–6.75)	-0.262	0.794
	Term				Term			
OSI^¥^ (AU)	LPs	0.55 (0.41–0.68) 0.48 (0.41–0.69)	-0.478	0.633	LPs	0.28 (0.20–0.44) 0.38 (0.19–0.46)	-0.439	0.661
	Term				Term			

Data are stated as median with interquartile range values

^*^LPs = Late Preterm

^¥^Oxidative Stress Index, TAS: Plasma Total Antioxidant Status, TOS: Plasma total oxidant status. Mann–Whitney U test was used for data analyses

When we compared OS parameters before and after treatment, we found essential changes in TOS and OSI levels in the term group (*p* < 0.001, *p* = 0.001 respectively). We found important changes in TAS, TOS and OSI levels in the late preterm group, as in the term group (*p* = 0.012, *p* < 0.001, *p* < 0.001, respectively). It was found that the disulfide level, which was 26.2 (19.2–31.7) before the treatment, decreased to 19.5 (15.5–28.75) after the treatment (*p* = 0.001) in late preterm group. After CPAP treatment, significant changes were found in the disulfide/native thiol, disulfide/total thiol and native thiol/total thiol ratio as an indicator of the decrease in OS in the late preterm group (*p* = 0.029, *p* = 0.022, *p* = 0.019 respectively) (Table 3). We did not find any correlation between Downes/APGAR scores and OS parameters.

**Table 3:** Evaluation of  oxidative stress parameters before and after CPAP treatment in late preterm and term group

Parameters	Late Preterm					Term				
	**Neg**^**£**^** Pos Ties**	**Mean Rank**	**Sum of Rank**	**Z**	**p**	**Neg Pos Ties**	**Mean Rank**	**Sum of Rank**	**Z**	**p**
Native thiol (µmol/L)	27	25.83	697.50	-0.077	0.938	15	13.90	208.50	-0.195	0.846
	25	27.22	680.50			14	16.18	226.50		
	0					0				
Total thiol (µmol/L)	28	26.3	736.50	-0.433	0.665	17	13.41	228	-0.227	0.820
	24	26.73	641.50			12	17.25	207		
	0					0				
Disulfide (µmol/L)	35	28.07	982.50	-3.331	**0.001**	20	14.50	270	-1.32	0.245
	15	19.50	292.50			9	12.44	165		
	2					0				
Disulfide/Native thiol (%)	34	27.31	928.50	-2.181	**0.029**	19	15.68	256	-1.642	0.083
	18	24.97	449.50			10	11.90	179		
	0					0				
Disulfide/Total thiol (%)	34	27.66	940.5	-2.290	**0.022**	19	15.19	240	-1.381	0.187
	18	24.31	437.5			10	12.10	185		
	0					0				
Native thiol/Total thiol (%)	34	24	432	-2.341	**0.019**	10	9.60	96	-2.627	**0.009**
	18	27.82	946			19	17.84	339		
	0					0				
TAS^*^ (mmol Trolox equiv. /lt)	20	20.68	413.50	-2.509	**0.012**	12	12.67	152	-1.417	0.157
	32	30.14	964.5			17	16.65	283		
	0					0				
TOS^π^ (µmol H_2_O_2_ equiv. /lt)	44	27.38	1204.5	-5.076	** < 0.001**	23	16.24	373.50	-3.384	** < 0.001**
	7	17.36	121.5			5	6.50	32.50		
	1					1				
OSI^¥^ (AU)	42	29.21	1227	-4.90	** < 0.001**	22	16.98	373.50	-3.37	**0.001**
	10	15.1	151			7	8.79	61.50		
	0					0				

^*^TAS Plasma Total Antioxidant Status

^π^*TOS* Plasma total oxidant status, *¥OSI* oxidative stress index, £ = *Neg* Negative, *Pos* Positive, Wilcoxon Test was used for evaluation

## Discussion

This is the first study in the literature to examine the dynamic changes of thiol/disulfide homeostasis in late preterm infants with TTN. In this study, TAS value, which is an indicator of antioxidant capacity, increased after treatment in late preterm group (*p* = 0.012); TOS capacity decreased in support of this result in both groups (*p* < 0.001, *p* < 0.001, respectively). Significant changes in disulfide levels, disulfide/native thiol, disulfide/total thiol and native/total thiol ratios in the late preterm group (*p* = 0.001, *p* = 0.029, *p* = 0.022, p = 0.019, respectively) were among the most important findings in the study.

The development of organs and vital systems such as the central nervous system continues from conception to adulthood in newborns and children [12]. The newborn and infancy period, where this growth is affected more rapidly and more by external factors, is very important in childhood growth and development. As a result of physical, biochemical factors and diseases encountered in early childhood, especially in neonatal period, the body's own OS balance is disrupted and causes many complications and morbidity [12, 13]. The important studies have been carried out recently regarding the effect of thiol disulfide homeostasis, a new marker of the oxidative system, on diseases in the pediatric period [6, 8, 9, 13].

Premature infants, in whom the antioxidant capacity is insufficient and the balance between oxidant/antioxidant metabolites can change rapidly towards oxidative damage, are more affected by this situation [14, 15]. Regarding this situation, researchers have examined the relationship of OS markers such as melatonin, glutathione peroxidase, SOD, and N-acetylcysteine in diseases such as hyperbilirubinemia, pneumonia, and RDS in the neonatal period [13, 16, 17]. It has been shown that increased OS status according to antioxidant capacity in critical diseases such as RDS causes mortality and morbidity in newborns [18]. In studies conducted in newborns exposed to hypoxia, it has been shown that the increase in OS markers such as Hx, Xa, TH and AOPP is associated with the degree of hypoxia [15, 19, 20]. OS studies on RDS, which is one of the causes of mortality in premature newborns, have shown that ROS products such as MDA, protein carbonyls, and 7,8-OHdG increase more than in healthy infants [15, 21]. It has been stated that the OS products that formed in the change of this balance play an important role in the pathogenesis of RDS. It has been shown that endotracheal surfactant treatment applied in patients with RDS reduces OS and increases antioxidant products [18, 22]. It was determined that the disulfide/native thiol disulfide/total thiol ratios were higher than the control group in the study in which the relationship between OS and TTN with term infants was evaluated [5]. The decreased disulfide level, disulfide/native thiol, disulfide/total thiol ratios (*p* = 0.001, *p* = 0.029, *p* = 0.022) after treatment compared to term infants, are one of the important results showing that TTN increases OS more in late preterm infants. Respiratory workload develops due to the inability to clean the intrauterine alveolar fluid after birth, the inability to provide gas exchange and the need for O_2_. We thought that the outflow of fluid in the alveoli and the initiation of gas exchange in the alveoli with CPAP treatment were associated with the reduction of oxidative stress.

Conditions in which respiratory workload increases in newborns such as RDS, BPD, congenital pneumonia, and TTN which appear in the process that begins with birth, increase the stress load by changing the oxidative balance [16, 23]. Öktem et al. reported in their study on newborns with pneumonia, the increase in thiol levels after treatment showed that OS decreased [9]. Newborns with BPD are exposed to ROS, hypoxia, and mechanical ventilation, in addition to infection and inadequate lung capacity. As a result of these exposures, decreased antioxidant capacity and increased oxidative products have been associated with BPD [13, 24]. In another study on BPD, 8-hydroxy-2-deoxyguanosine (8-OHdG) levels in serum and tracheal aspirate samples of preterm infants were found to be higher on the 1st and 28th days after birth [25]. Protein carbonyl levels were found to be higher in preterms who developed BPD, and they associated the increase in protein carbonyl levels, which is a parameter of OS, with the pathogenesis of the disease [15, 16]. TTN, which causes respiratory distress in newborns in the early period, increases the stress situation in the newborn with the progression of the clinical findings of the disease [26, 27]. Erdal et al. in their study in newborns with TTN, they reported that TOS and OSI levels increased significantly compared to the control group [5]. In parallel with the studies in the literature on respiratory problems in the neonatal period, the decrease in TOS and OSI, which are indicators of oxidant capacity, in both groups after TTN treatment in our study shows that TTN increases OS in newborns. Moreover, the significant increase in TAS (p = 0.012) and native thiol/total thiol levels (*p* = 0.019) and decrease in the disulfide levels (*p* = 0.001) after the treatment in the late preterm group show that OS affects the late preterms more specifically.

## Conclusion

CPAP treatment in TTN reduced the increased OS burden associated with TTN in neonates. It has been shown that late preterms with TTN are more affected by OS and good response is obtained with CPAP treatment. Thiol/disulfide may be preferred as an indicator of OS in patients with late preterm TTN. The clinical substantial of this study is that measurement of dynamic thiol disulfide levels in TTN patients will provide the clinician with an important perspective on OS in the management of the disease. Our study has been a pioneering study that can contribute to the literature in randomized controlled studies and cohort studies on TTN in newborns.

## Data Availability

All data generated or analysed during this study are included in this published article.

## Abbreviations

TTN: Transient tachypnea of the newborn
OS: Oxidative stress
CPAP: Continuous airway positive pressure
TAS: Plasma total oxidant status
TOS: Plasma total oxidant status
OSI: Oxidative stress index
ENaC: Amiloride-sensitive Na^+^ channels
NICU: Neonatal intensive care unit
Hx: Hypoxanthine
Xa: Xanthine
MDA: Malondialdehyde
TH: Total hydroperoxide
AOPP: Advanced oxidation protein products
BPD: Bronchopulmonary dysplasia
8-OHdG: 8-Hydroxy-2’-deoxyguanosine
CBC: Complete blood count
RDS: Respiratory distress syndrome
SH: Sulfhydryl group
ABTS^+^: 2,2′-Azino-bis (3-ethylbenzthiazoline-6-sulfonic acid
